# Evolution of whole-body enantiomorphy in the tree snail genus *Amphidromus*

**DOI:** 10.1111/j.1420-9101.2006.01246.x

**Published:** 2007-03

**Authors:** C SUTCHARIT, T ASAMI, S PANHA

**Affiliations:** * Department of Biology, Faculty of Science, Chulalongkorn University Bangkok, Thailand; † Department of Biology, Shinshu University Matsumoto, Japan; ‡ PRESTO, Japan Science and Technology Agency Kawaguchi, Japan

**Keywords:** asymmetry, Camaenidae, chirality, gastropod, mtDNA phylogeny, pulmonate

## Abstract

Diverse animals exhibit left–right asymmetry in development. However, no example of dimorphism for the left–right polarity of development (whole-body enantiomorphy) is known to persist within natural populations. In snails, whole-body enantiomorphs have repeatedly evolved as separate species. Within populations, however, snails are not expected to exhibit enantiomorphy, because of selection against the less common morph resulting from mating disadvantage. Here we present a unique example of evolutionarily stable whole-body enantiomorphy in snails. Our molecular phylogeny of South-east Asian tree snails in the genus *Amphidromus* indicates that enantiomorphy has likely persisted as the ancestral state over a million generations. Enantiomorphs have continuously coexisted in every population surveyed spanning a period of 10 years. Our results indicate that whole-body enantiomorphy is maintained within populations opposing the rule of directional asymmetry in animals. This study implicates the need for explicit approaches to disclosure of a maintenance mechanism and conservation of the genus.

## Introduction

Diverse groups of animals exhibit asymmetry in internal structure. Visceral asymmetry generally indicates the direction of primary asymmetry, which corresponds to the left–right polarity in early development (Crampton, 1894; Wood, 1997; Levin, 2005; Hozumi *et al.*, 2006). Some other body parts such as crossbill mandibles (Benkman, 1996) and lobster claws (Govind, 1989) display the secondary asymmetry, which is generally independent of the primary asymmetry in the polarity. For example, the asymmetry of the digestive tract is not reversed between the enantiomorphs of flounder eyes (Policansky, 1982) or of bivalve shells (Odhner, 1919). In a variety of animals, mutation in molecular pathways controlling the direction of the primary asymmetry is known to produce whole-body enantiomorphs (Crampton, 1894; Félix *et al.*, 1996; Levin, 2005; Hozumi *et al.*, 2006). However, the reversal of internal asymmetry has gathered little attention except for vertebrates and some others, compared with the variation of the presumable secondary asymmetry in external traits (Palmer, 1996, 2004, 2005). Possible reversal of the primary asymmetry would not be recognized unless developmental or visceral asymmetry is identified, even if external enantiomorphs are found such as in barnacles (Yusa *et al.*, 2001). Nevertheless, it is clear that the primary asymmetry does not generally exhibit chiral dimorphism (enantiomorphy), except for few groups (Félix *et al.*, 1996; Asami *et al.*, 1998).

Considering the general rule of directional asymmetry in animal development, gastropods are a unique group in exhibiting a diverse array of mirror image species. Existence of dextral and sinistral species in phylogenetically independent clades indicates that left–right reversal of whole-body asymmetry has repeatedly evolved in gastropods (Vermeij, 1975, 1993; Gittenberger, 1988; Robertson, 1993; Asami *et al.*, 1998). The left–right polarity of spiral cleavage corresponds to the direction of torsion and visceral asymmetry developing later, at least in pulmonates (Crampton, 1894; Camey & Verdonk, 1970; Meshcheryakov & Beloussov, 1975). Sinistral and dextral snails are thus whole-body enantiomorphs reversed in the primary asymmetry. However, they cannot necessarily be distinguished by the coiling direction. Dextral species in the primary asymmetry coil clockwise in orthostrophic groups but anticlockwise in hyperstrophic groups (Vermeij, 1975; Robertson, 1993), because the two groups form helices in opposite directions along the coiling (dorsoventral) axis. Thus, the coils of gastropods exhibit the secondary asymmetry, independent of the primary asymmetry in polarity.

Sinistral species have evolved in terrestrial pulmonates nearly 10 times as often as in aquatic gastropods (Robertson, 1993), although dextral species still remain overwhelmingly predominant in most groups (Vermeij, 1975; Gould *et al.*, 1985). In four phylogenetically independent superfamilies of pulmonates (Wade *et al.*, 2006), either the sinistral or the dextral is dominant in maternal inheritance (Toyama, 1913) at a single nuclear locus (Sturtevant, 1923; Boycott *et al.*, 1930; Degner, 1952; Murray & Clarke, 1976; Ueshima & Asami, 2003). In *Lymnaea*, the sinistral is recessive to the dextral because only the latter allele produces the chiral determinant (Freeman & Lundelius, 1982). This simple genetic basis of chiral reversal may have allowed relatively frequent evolution of mirror image species in snails (Gittenberger, 1988; Orr, 1991; van Batenburg & Gittenberger, 1996; Ueshima & Asami, 2003).

However, enantiomorphy is seldom found within populations of snails (Johnson, 1982; Asami *et al.*, 1998). Because sinistrals and dextrals are whole-body enantiomorphs which are reversed in the genital opening on the body side and mating behaviours as well, copulation between the morphs is physically difficult (Lipton & Murray, 1979; Johnson, 1982; Gittenberger, 1988; Asami *et al.*, 1998; Ueshima & Asami, 2003). Thus, in populations of internally fertilizing gastropods, the less frequent morph should suffer disadvantage in mating with the common morph, resulting in positive frequency-dependent selection (Johnson, 1982). Before its extinction in the wild (Murray *et al.*, 1998), the Polynesian tree snail *Partula suturalis* exhibited enantiomorphy in narrow areas as a result of dispersal from stably monomorphic populations of sinistrals and dextrals, which is consistent with acute frequency-dependent selection against enantiomorphy (Johnson, 1982, 1987; Johnson *et al.*, 1993). Enantiomorphy has also been recorded in at least 13 genera over eight superfamilies (Asami *et al.*, 1998), but no example of enantiomorphy has been shown to be maintained within populations.

Despite the general absence of enantiomorphy in snail populations, three tree snail genera *Achatinella* (Welch, 1938, 1942, 1954), *Amphidromus* (Sutcharit & Panha, 2006b) and *Partula* (Crampton, 1916, 1932) are known for frequent enantiomorphy in multiple species. These genera are phylogenetically independent (Wade *et al.*, 2006) and thus suggest independent evolution of enantiomorphy within species, contrary to the general absence of transition from directional asymmetry to enantiomorphy in animals (Palmer, 2004). However, it has not even been questioned whether enantiomorphy in multiple species is monophyletic. Little is known of whether and how enantiomorphs coexist within populations, except for a few cases in *Partula* (Johnson *et al.*, 1993). The genetic basis of enantiomorphy is known in only a few species of *Partula* (Murray & Clarke, 1976, 1980). Most species of *Achatinella* and *Partula* have recently become extinct (Hadfield, 1986; Cowie, 1992; Hadfield *et al.*, 1993; Murray *et al.*, 1998). Only South-east Asian tree snails of the genus *Amphidromus* now allow us explicit approaches to the evolution of whole-body enantiomorphy in multiple species in the wild.

*Amphidromus* (Camaenidae) is a genus of tree snails which occur over most of South-east Asia and surrounding areas (Laidlaw & Solem, 1961; Sutcharit & Panha, 2006b). Variation in the complex shell-colour patterns within and between populations has long confused the traditional taxonomy of *Amphidromus* (Sutcharit & Panha, 2006b). Thus the conchological taxonomy of *Amphidromus* has been subjected to repeated revisions. Of over 300 nominal species have been proposed, only 85 species remain but these still require thorough reviews incorporating the outcome of biological and molecular approaches (Sutcharit & Panha, 2006b). Currently the genus is divided into two subgenera, *Amphidromus (Amphidromus)* and *A. (Syndromus)*, which are distinct in shell size and colour pattern and in genital morphology (Laidlaw & Solem, 1961; Sutcharit & Panha, 2006b). The subgenus *Amphidromus* has a large shell (35–75 mm in height; 20–40 mm in width) and a long epiphallic caecum, whereas *Syndromus* has a small shell (20–40 mm in height; 10–25 mm in width) and a short epiphallic caecum. The subgenus *Amphidromus* contains 32 species, predominantly dimorphic for chirality (enantiomorphic), with the exception of four dextral taxa, *A. givenchyi*, *A. protania*, *A. schomburgki dextrochlorus* and *A. inversus annamiticus* and one sinistral taxon, *A. atricallosus classiaris*. In contrast, *Syndromus* includes all 44 sinistral species with one exceptionally enantiomorphic species, *A. glaucolarynx* (Pilsbry, 1900; Laidlaw & Solem, 1961; Solem, 1965; Richardson, 1985; Sutcharit & Panha, 2006a,b).

Considering the dextral monomorphism of possible sister taxa (Pilsbry, 1901; Gude, 1914; Laidlaw & Solem, 1961; Wade *et al.*, 2006), the ancestor of the genus *Amphidromus* most likely diverged from a dextral species, assuming the monophyly of the genus. Therefore, the unusual presence of every possible character state of handedness, sinistral and dextral monomorphism and enantiomorphy, in multiple species in the genus prompts a fundamental evolutionary question: how have sinistrality and enantiomorphy evolved from the presumably dextral ancestor? The answer should provide useful insights into evolutionary processes behind the complex diversity of whole-body handedness. Their relationships, however, have not been examined by means of molecular phylogeny. Enantiomorphs of *A. inversus albulus* have been shown to coexist on a Malaysian island (Schilthuizen *et al.*, 2005; Sutcharit & Panha, 2006a). However, little is known of the generality or stability of enantiomorphy within or between populations.

We conducted both mtDNA phylogeny and field surveys to elucidate evolutionary processes underlying such a complex pattern of chiral evolution in the genus *Amphidromus*. Here we demonstrate that the whole-body enantiomorphy of *Amphidromus* is evolutionarily stable and persists within populations in general.

## Materials and methods

### mtDNA samples

**Fig. 1:**
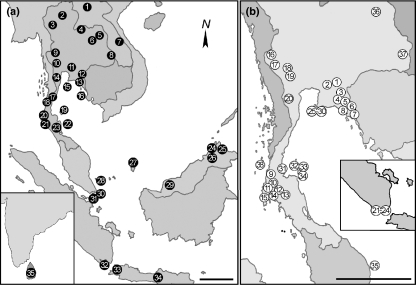
Sampling localities. (a) Sampling localities for an mtDNA phylogeny of the genus *Amphidromus*. Numbers correspond to those in Table 1, Fig. 2 and Table S1. The inset indicates a locality on Sri Lanka. (b) Localities for surveys of spatial and temporal variations of enantiomorph frequencies in *A. atricallosus*, *A. glaucolarynx*, *A. inversus* and *A. givenchyi*. Numbers correspond to those in Tables 2 and 3 and Table S2. The inset indicates localities in south-eastern Sumatra. Scale bars indicate 200 km. Approximate areas searched for snails are available in Table S1.

We used foot tissues of 65 individuals of 11 taxa in eight species of the subgenus *Amphidromus* and 33 individuals of nine species in the subgenus *Syndromus* (Table 1), collected from 35 localities in South-east Asia (Fig. 1a, see Table S1). As the outgroup taxa, we used *Camaena illustris*, *Chloritis siamensis* and *Beddomea albizonatus*, which have been suggested as basal to the genus *Amphidromus* (Pilsbry, 1901; Gude, 1914; Laidlaw & Solem, 1961). We examined the type specimens of all the taxa involved in this study and identified specimens based on Pilsbry (1900), Laidlaw & Solem (1961), Solem (1965) and Sutcharit & Panha (2006b). We amplified a fragment of mtDNA coding 16S rRNA by PCR with primers 16Scs1 5′-AAACATACCTTTTGCATAATGG-3′ (Chiba, 1999) and 16Sbd1 5′-CTGAACTCAGATCATGTAGG-3′. We aligned nucleotide sequences using clustal w 1.4 (Thompson *et al.*, 1994) and confirmed them by eye. We excluded 10 regions of ambiguous alignment from the final data set, which reduced the sequence alignment to 845-bp regions. For phylogenetic analysis, we used sequences of 780 bp, deposited in DNA Data Bank of Japan (DDBJ) (Table 1).

**Fig. 2:**
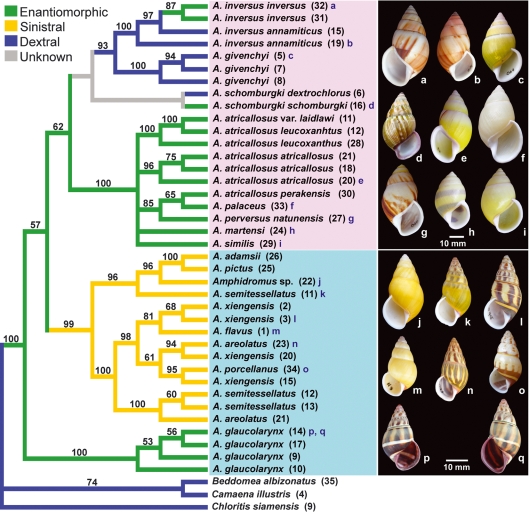
Maximum parsimony mtDNA phylogeny of the genus *Amphidromus* based on partial 16S rDNA sequences with representatives of the genera *Camaena*, *Chloritis* and *Beddomea* as outgroup. Numbers in parentheses indicate sampling localities (Table 1, Fig. 1a, see Table S1). Letters following these numbers correspond to the shells illustrated to the right. The background colours indicate the currently recognized subgenera, *Amphidromus* and *Syndromus*. The cladogram is a strict consensus of 12 parsimonious trees, based on 469 bp informative sites and generated using the heuristic option. Bootstrap probabilities above 50% in 1000 replicates are shown at the nodes.

**Table 1:** Specimens examined for molecular phylogeny.

Taxon	Locality	*n*	16S rDNA accession number
*A. (A.) atricallosus atricallosus* (Gould, 1843)	18, 20, 21	5D, 6S	AB112365, AB112393–94
*A. (A.) atricallosus leucoxanthus* (von Martens, 1864)	11, 12, 28	5D, 1S	AB112369, AB112392, AB112395
*A. (A.) atricallosus perakensis*Fulton, 1901	30	4D, 1S	AB112368
*A. (A.) inversus inversus* (Müller, 1774)	31, 32	6D, 2S	AB112367, AB112400
*A. (A.) inversus annamiticus* (Crosse & Fischer, 1863)	15, 19	15	AB112366, AB112391
*A. (A.) schomburgki schomburgki* (Pfeiffer, 1860)	16	1D	AB112396
*A. (A.) schomburgki dextrochlorus* Sutcharit & Panha, 2006	6	2D	AB112373
*A. (A.) perversus natunensis* Fulton, 1896	27	2D	AB112375
*A. (A.) palaceus* (Mousson, 1848)	33	1D, 2S	AB112374
*A. (A.) martensi* Boettger, 1894	24	1D	AB112376
*A. (A.) similis*Pilsbry, 1900	29	2D, 2S	AB112371
*A. (A.) givenchyi* Geret, 1912	5, 7, 8	7	AB112372, AB112398–99
*A. (S.) pictus* Fulton, 1896	25	1	AB112381
*A. (S.) adamsii* (Reeve, 1848)	26	1	AB112370
*A. (S.) areolatus* (Pfeiffer, 1861)	21, 23	6	AB112387, AB112405
*A. (S.) xiengensis* Morlet, 1891	2, 3, 15, 20	9	AB112377, AB112397, AB112401–02
*A. (S.) semitessellatus* Morlet, 1884	11, 12, 13	6	AB112379, AB112403–04
*A. (S.) flavus* (Pfeiffer, 1861)	1	1	AB112386
*A. (S.) porcellanus* (Mousson, 1848)	34	3	AB112380
*Amphidromus (S.)* sp.*	22	1	AB112378
*A. (S.) glaucolarynx* (Dohrn, 1861)	9, 10, 14, 17	4D, 1S	AB112382–85
Outgroup			
*Beddomea albizonatus* (Reeve, 1849)	35	1	AB112388
*Camaena illustris* (Pfeiffer, 1863)	4	1	AB112389
*Chloritis siamensis* Möllendorff, 1902	9	2	AB112390

Locality numbers correspond to those in Figs 1a and 2 and Table S1. *n* indicates the number of dextrals (D) and/or sinistrals (S) examined.

* An undescribed species.

**Table 2:** Enantiomorph frequencies of living snails of all ages in six *Amphidromus* taxa.

Species	Locality	Year	Dextral	Sinistral	Percentage of sinistral	Within taxon
*A. atricallosus leucoxanthus*	1	1994	20	14	0.412	0.001
	2	2000	18	1	0.052**	
	3	2004	14	20	0.588	
	4	2003	5	1	0.167	
	5	2002	38	25	0.397	
	7	2004	65	6	0.085***	
*A. atricallosus atricallosus*	9	2003	5	3	0.375	0.001
	10	2003	8	2	0.200	
	11	2000	17	24	0.585	
	13	2003	54	44	0.449	
	14	2004	3	5	0.625	
	15	2003	2	18	0.900**	
*A. atricallosus classiarius*	38	1999–2003	0	123	1.000	
*A. glaucolarynx*	16	2002	5	32	0.865***	0.001
	17	2002	8	6	0.429	
	19	2000	1	5	0.833	
	20	2004	14	5	0.263	
*A. inversus inversus*	21	1994	11	136	0.925***	0.024
	22	1994	4	127	0.969***	
	23	1994	4	62	0.939***	
	24	1994	7	34	0.829***	
*A. inversus albulus*	35	2004	69	167	0.708***	
*A. inversus annamiticus*	25	2001–2004	26	0	0.000	
	26	1998–2003	60	0	0.000	
	27	1998–2003	75	0	0.000	
	28	2001	31	0	0.000	
	29	2000–2001	65	0	0.000	
	30	2000–2001	11	0	0.000	
	31	2003	28	0	0.000	
	32	2002	7	0	0.000	
	33	2000	27	0	0.000	
	34	2002	46	0	0.000	
*A. givenchyi*	36	2002	31	0	0.000	
	37	2000–2003	12	0	0.000	

Locality numbers correspond to those in Fig. 1b and Table S2.

** and *** indicate significant deviations from 50% after Bonferroni correction for multiple comparisons at the probabilities 0.05, 0.01 and 0.001 respectively. ‘Within taxon’ indicates significance of frequency variation among localities within each taxon. The sample of *A. atricallosus classiarius* includes 22 empty shells.

**Table 3:** Persistence of enantiomorphy in *Amphidromus atricallosus* and *A. glaucolarynx*.

Species	Locality	Year	Dextral	Sinistral	Percentage of sinistral	Temporal shift
*A. atricallosus leucoxanthus*	6	1996	12	5	0.294	0.666
		2001	28	22	0.440	
		2002	116	75	0.393*	
		2004	67	51	0.432	
	8	1994	3	4	0.571	0.001
		1996	16	2	0.111*	
		1999	36	9	0.2**	
		2001	137	24	0.149***	
		2002	281	24	0.079***	
		2003	5	4	0.444	
*A. atricallosus atricallosus*	12	1995	*21*	*35*	0.625	0.002
		1999	13	33	0.717	
		2001	250	560	0.691***	
		2002	208	318	0.605***	
		2003	81	134	0.623**	
		2004	32	32	0.500	
*A. glaucolarynx*	18	1994	*17*	*13*	0.433	0.272
		1995	*13*	*12*	0.480	
		1996	3	3	0.500	
		1998	7	3	0.300	
		1999	33	43	0.566	
		2000	30	49	0.620	

Locality numbers correspond to those in Fig. 1b and Table S2. Italic numbers indicate samples of empty shells.

*, ** and *** indicate significant deviations from 50% after Bonferroni correction for multiple comparisons at the probabilities 0.05, 0.01 and 0.001 respectively. Temporal shift indicates significance of frequency changes across the period of survey at each locality.

### Phylogenetic analysis

We conducted maximum parsimony (MP), maximum likelihood (ML) and neighbor-joining (NJ) analyses using paup* 4.0 (Swofford, 2002). The NJ method (Saitou & Nei, 1987) was applied on the basis of a pairwise matrix of the distance from Jukes–Cantor (Jukes & Cantor, 1969) and Kimura 2-parameter (Kimura, 1980). For the MP analysis, we applied equal weighting and a heuristic search option with tree bisection reconnection branch-swapping and 100 random additions. We assumed the transition : transversion bias as 2 : 1 in all analyses according to the observed ratio in the ingroup (transition/transversion = 1.59). We used the bootstrap probability (Felsenstein, 1985) to test the reliability of each node based on 1000 replicates for MP and NJ methods, and 100 replicates for the ML method.

### Field survey

We surveyed phenotype frequencies of six taxa, *A. atricallosus*, *A. glaucolarynx*, *A. inversus albulus*, *A. inversus inversus*, *A. inversus annamiticus* and *A. givenchyi*, in 38 localities within determined areas mostly at night using flashlights (Fig. 1b, see Table S2). In several places, we surveyed along a trail because of the physical difficulties of surveying in quadrats. We repeated surveys of enantiomorphic populations in four localities from 1994 to 2004. We avoided using empty shells to assess enantiomorphy because it cannot be certain when they were living, except in the case of the oldest records at localities involved in periodical surveys. We tested the statistical significance of differences in enantiomorph frequency by the Fisher exact test. Tree snails of the subgenus *Amphidromus* lay eggs in the canopy between living leaves held attached to each other by mucus (Sutcharit & Panha, 2006b). We collected three clutches from the canopy in Chanthaburi, Thailand, where only *A. atricallosus* has been found in the subgenus. The large size of eggs, covered with leaves in a peculiar manner, indicated that they were laid by *A. atricallosus*.

## Results

### Sequence variation

In the aligned partial sequences of the 16S rRNA gene, 55.5% of 845 bp was variable. In the average base frequency of all taxa, A and T were higher (36.5% and 30.9% respectively) than C and G (13.7% and 18.9% respectively). The base frequencies did not differ significantly across taxa (chi-square test implemented in paup*, *P* = 0.99). Sequence divergence between the genus *Amphidromus* and each of the outgroups, *Beddomea*, *Camaena* and *Chloritis* ranged from 32.6% to 38.2%. Divergence between the subgenera *Amphidromus* and *Syndromus* ranged from 22.3% to 29.7%. Within the subgenera, it was between 8.2% and 24.0% in *Amphidromus*, and between 0.4% and 29.3% in *Syndromus*. Divergence between subspecies ranged from 10.0% to 12.4% in *A. atricallosus* and from 1.8% to 5.0% in *A. inversus*. Within species of *Syndromus*, haplotypes varied in 5.2–8.8% of base pairs in *A. glaucolarynx*, 2.6–10.3% in *A. xiengensis*, 0.7–22.5% in *A. semitessellatus*, and 17.5% in *A. areolatus*. Sinistral and dextral morphs differed from each other at only a few base pairs in each of six taxa, where samples of both morphs were available from the same localities. Sequence differences between the morphs were equivalent to those within the morphs.

### Phylogeny

The phylogeny reconstructed by MP, NJ and ML methods were congruent with one another (Fig. 2, see Figs S1 and S2). Most branching nodes were supported with reliably high bootstrap values for inference of evolutionary history based on the topology. The genus *Amphidromus* is a monophyletic clade. Enantiomorphic *A. glaucolarynx* splits most basally from the rest of the members. The cluster exclusive of *A. glaucolarynx* divides into two groups: the subgenus *Amphidromus* and sinistral *Syndromus*. In the former clade, samples of *A. atricallosus* did not constitute a monophyletic cluster and suggested the presence of three lineages. In one of those, *A. atricallosus perakensis* from Singapore was clustered with *A. palaceus* from Java and *A. perversus natunensis* from Natuna Island, Indonesia. In contrast to the subgenus *Amphidromus*, the phylogeny of sinistral *Syndromus* shows little correspondence to the current taxonomy. Specimens of *A. semitessellatus* and *A. areolatus* fall into two different clades. The three haplotypes of *A. xiengensis* cluster with three different species.

The present molecular phylogeny resolved complex chiral diversity into discrete patterns of cladogenesis. Despite the conservative expectation of dextral ancestry, the results indicate that enantiomorphy is the ancestral state of chirality in the genus. Incipient secondary enantiomorphy was detected within *A. inversus*, which is derived from a dextral ancestor. Although additional information is required to resolve the ancestral handedness of *A. schomburgki*, the current result indicates that either enantiomorphy or dextrality is secondarily derived. In contrast, the clade of sinistral *Syndromus* shows notable stability of sinistral monomorphism, indicating sinistrality has evolved at least twice including sinistral *A. atricallosus classiarius* in the history of the genus.

### Coexistence of enantiomorphs

Our field surveys of enantiomorphic *Amphidromus* found no evidence of monomorphic populations (Tables 2 and 3). Instead, enantiomorphs consistently coexist in all 25 localities where *A. atricallosus*, *A. glaucolarynx* or *A. inversus* was found. In contrast, we found no reversed variant but only dextrals in populations of *A. inversus annamitticus* and *A. givenchyi*. Our repeated surveys demonstrate that enantiomorphy continuously persists over at least 10 years in two subspecies of *A. atricallosus* (Table 3). However, morph frequencies significantly varied in time and space within species and also between species as follows. Morph frequencies in living snails differed between localities within every enantiomorphic taxon surveyed (contingency table, Fisher exact test, *A. inversus inversus*, *P* = 0.024; other taxa, *P* ≤ 0.001). Sinistral frequencies did not differ between the two subspecies *A. atricallosus atricallosus* and *A. atricallosus leucoxanthus* (*U* = 8, *P* = 0.132). After the Bonferroni correction for multiple comparison between species, sinistral frequency was higher in *A. inversus inversus* than in *A. atricallosus* (*U* = 1, *P* < 0.05), but *A. glaucolarynx* showed no significant differences from *A. atricallosus* (*U* = 15, *P* = 0.316) or *A. inversus inversus* (*U* = 2, *P* = 0.114). During 10 years of survey, frequencies shifted in both subspecies of *A. atricallosus* (contingency table, Fisher exact test, *P* ≤ 0.002). In 16 of 44 enantiomorphic samples, frequencies were significantly biased towards one morph or the other (binomial tests with Bonferroni correction). Overall, however, there was no consistent bias to either the sinistral or dextral morph across the collection sites (Wilcoxon signed-ranks test, *U* = 0.342). Therefore, in the genus *Amphidromus*, whole-body enantiomorphs generally coexist within populations, and the morph frequency is spatially and temporarily variable but exhibits no local fixation.

## Discussion

### Evolutionary stability of enantiomorphy

Our mtDNA phylogeny has shown that the genus *Amphidromus* has probably retained whole-body enantiomorphy within species throughout the radiation of diverse species since the earliest ancestor became enantiomorphic. In that sense, enantiomorphy is evolutionarily stable in *Amphidromus*, contrary to the prevailing view that chiral monomorphism for the primary asymmetry is the rule in snails as well as in other animals.

The nucleotide substitution rates available for pulmonate mitochondrial rDNA suggest that the enantiomorphy of *Amphidromus* could have originated as early as the late Eocene [35 million years ago (Ma)], based on the most conservative estimate (Douris *et al.*, 1998), or as recently as the early Pliocene (2.5 Ma), based on the highest rate (Thacker & Hadfield, 2000). Therefore, *Amphidromus* species, which have approximately 2-year generation times, have probably retained the enantiomorphy over at least a million generations. Molecular phylogenies of the other two groups of enantiomorphic tree snails, *Achatinella* (Thacker & Hadfield, 2000; Holland & Hadfield, 2004) and *Partula* (Goodacre & Wade, 2001a,b), did not provide comparable information because of the recent extinction of many species (Hadfield, 1986; Cowie, 1992; Hadfield *et al.*, 1993; Murray *et al.*, 1998). However, they have shown nothing contrary to the evolutionary stability of enantiomorphy.

The secondary enantiomorphy of *A. inversus inversus* shows that enantiomorphy is not disappearing in the clade of *Amphidromus* but has evolved independently of the ancestral enantiomorphy, a finding that is supported by high bootstrap probabilities. Thus, the present results demonstrate that whole-body enantiomorphy has repeatedly evolved from dextral monomorphism, in contrast to the virtual absence of enantiomorphy derived from directional asymmetry in other animals (Palmer, 2004). Sinistrality has, however, been stable since fixation in the ancestor of *Syndromus*. A more comprehensive study including the rest of available taxa in the genus is needed to validate that the enantiomorphy of *A. glaucolarynx* and the cluster of *A. atricallosus* is ancestral but not derived recently.

The enantiomorphs did not differ from each other in mtDNA sequences within localities in the seven taxa examined, as expected from their reciprocal interchiral copulation which will be discussed below. It is also consistent with the prediction of gene flow on the assumption of the maternal inheritance (Toyama, 1913) of the primary asymmetry in *Amphidromus* (Johnson *et al.*, 1987, 1990). However, little is known of the genetic basis of enantiomorphy in *Amphidromus*. Hatchlings from three clutches we obtained near the locality 6, where 30–40% of snails are sinistral, were all identical in chiral phenotype: 94 and 66 dextrals and 69 sinistrals. These are consistent with the model of maternal inheritance determined by the dextral and sinistral alleles. To verify the present result on gene flow, larger samples from enantiomorphic populations should be examined using nuclear markers, or maternal inheritance of handedness determined by a nuclear gene needs to be validated.

### Coexistence and persistence in the field

The evolutionary stability of enantiomorphy, revealed by molecular phylogeny, does not by itself indicate that enantiomorphs coexist within populations of *Amphidromus*. In *Partula suturalis*, dextrals and sinistrals separately persisted in monomorphic populations as a result of reproductive character displacement (Johnson *et al.*, 1993). In the subgenus *Amphidromus*, however, interspecific sympatry necessary for the evolution of character displacement seen in *Partula* or *Isabellaria* (Uit de Weerd *et al.*, 2006) has not been found even in the numerous empty shells on the ground. Moreover, we found no example of monomorphic population in species that are known for enantiomorphy. The two morphs were commonly found together on the same trees in most of enantiomorphic populations. Thus, enantiomorphy in the subgenus *Amphidromus* does not result from the presence of sinistral and dextral monomorphic populations but indicates coexistence of the morphs within populations.

In our repeated surveys, enantiomorphy has continuously persisted over at least 10 years, several times as long as the approximate 2-year generation time. The morphs were both present with no exception in the 25 sites we surveyed for five enantiomorphic taxa. However, the morph frequencies exhibit a wide range of variation from 5% to 97% sinistrals, deviating significantly from 50% in many cases. They also significantly vary both spatially and temporally within most taxa examined. Thus, it remains unclear how regularly the morph frequency varies between enantiomorphic populations, where we have found substantial variation from 32.5% in *A. atricallosus leucoxanthus* to 91.6% in *A. inversus inversus* in the mean sinistral frequency. The size and shape of the area surveyed varied among the sites because of physical conditions such as topography, vegetation, and habitat fragmentation. Surveys along trails, for example, might have involved multiple panmictic demes. The environment also varies across forests, farms, and residential areas where we searched for snails. Thus, the effects of unknown site-dependent factors on morph frequency might have been confounded, although no plausible environmental correlate with enantiomorph frequency has been discovered.

We found that *A. atricallosus* achieves simultaneous reciprocal copulation between enantiomorphs as well as within enantiomorphs in survey sites in Suratthani and Chanthaburi, Thailand (see Fig. S3). These observations are against two general expectations. First, high-spired species mate nonreciprocally by shell mounting, whereas low-spired species perform simultaneous reciprocal copulation face to face, in general. Secondly, reciprocal copulation between enantiomorphs is known to be seldom possible (Gittenberger, 1988; Asami, 1993; Asami *et al.*, 1998; Ueshima & Asami, 2003). Furthermore, reciprocal copulation was confirmed even between the morphs attached side by side to a tree trunk, both facing up in the same direction, with the sinistral snail to the left of the dextral snail (see Fig. S3). In this posture, genital openings are further away from each other than in the ordinary face-to-face trials between the morphs. These observations suggest that enantiomorphs of *A. atricallosus* can ordinarily mate with each other, although the relative frequency of interchiral mating needs to be quantified. *Amphidromus  inversus albulus* performs similar interchiral copulation (Schilthuizen & Davison, 2005). These enantiomorphic species of the subgenus *Amphidromus* extend the penis without close matching of genital openings between partners. In contrast, *A. xiengensis*, one of the sinistral species of *Syndromus*, copulates tightly as is typical of chirally monomorphic snails (see Fig. S3; Asami *et al.*, 1998). This type of tight joint and exactly symmetric positioning between partners are not possible between the opposite handedness and thus should cause stringent frequency-dependent selection for chiral monomorphism. These contrasting patterns of copulation behaviours between the subgenera may be attributable to their differences in epiphallic caecum, which is substantially longer in the subgenus *Amphidromus* than in *Syndromus*.

### Maintenance of enantiomorphy

The present outcome of molecular phylogeny and field surveys support the hypothesis that enantiomorphs coexist within populations through a particular mechanism that prevents stochastic fixation. The enantiomorphy of *A. inversus inversus* on all four of the Indonesian islands where they were sampled, each island smaller than 200 m in diameter, rules out persistence by concurrent gene flow in a metapopulation structure. Schilthuizen *et al.* (2005) has also rejected a similar process in *A. inversus albulus*. In contrast, no sinistral specimen has been recorded in *A. inversus annamiticus*, which occurs in continental Indochina and differs in colour patterns of the shell apex (protoconch) and upper spire from the other two subspecies (Fig. 2; Sutcharit & Panha, 2006a,b). Although the current samples of *A. inversus annamiticus* were mostly from Thai islands, dextral monomorphism is not simply attributable to random genetic drift in insular populations, because our phylogeny indicates that those dextral populations are not derived but ancestral to the enantiomorphic populations. Further, monomorphism is not associated with insular populations but has also been confirmed in every collection record of continental taxa such as *A. givenchyi* and *A. schomburgki dextrochlorus*.

South-east Asian snakes of the genus *Pareas* are known to feed exclusively on snails extracting the soft body from the shell (Götz, 2002; Hoso & Hori, 2006). If their predation is biased for the commoner enantiomorph and a chief determinant of morph frequency, the two morphs may be maintained equally frequently through negative frequency-dependent selection. However, 36% of the present cases significantly deviate from the expected 1 : 1 ratio of the morphs (Tables 2 and 3). Further, those deviations do not seem to result from frequency oscillation, which has been observed in the enantiomorphy of fish under frequency-dependent predation (Hori, 1993; Nakajima *et al.*, 2004). Some snail-eating aquatic predators are known to specialize in the dextral majority of prey (Inoda *et al.*, 2003; Dietl & Hendricks, 2006). Unless handedness-dependent predation occurs for both enantiomorphs, however, enantiomorphy could not be maintained by predation. Little is known of predators and their variability in handedness, and thus their possible roles for the maintenance need to be investigated.

If interchiral mating is frequent, enantiomorphs could long persist in populations under relaxed selection against the less common morph (Johnson, 1982; van Batenburg & Gittenberger, 1996; Asami *et al.*, 1998). However, maintenance of enantiomorphy requires interchiral mating to be more successful than intrachiral mating, assuming that the enantiomorphy of *Amphidromus* has a similar genetic basis to those known in other pulmonates. The long epiphallic caecum, typical of the subgenus *Amphidromus*, may be necessary for reciprocal interchiral copulation. However, the length of epiphallic caecum does not explain enantiomorphy because *A. inversus annamiticus*, *A. givenchyi* and *A. schomburgki dextrochlorus* also have a long epiphallic caecum but are all monomorphic, while enantiomorphic *A. glaucolarynx* has a much shorter epiphallic caecum than those dextral taxa. To disclose a mechanism responsible for the maintenance of enantiomorphy, possible handedness-dependent survival and/or reproductive success need to be examined.

Whole-body enantiomorphy displayed by *Amphidromus* may also have evolved in the Hawaiian and Polynesian tree snails *Achatinella* and *Partula* respectively. They might have led us to mechanistic approaches to the maintenance of enantiomorphy that have evolved in phylogenetically independent clades (Wade *et al.*, 2006). However, most members of *Achatinella* and *Partula* are extinct or highly threatened. It demonstrates the general susceptibility of tropical tree snails to habitat destruction, predation by introduced animals and over-collecting by shell collectors (Hadfield, 1986; Cowie, 1992; Hadfield *et al.*, 1993; Murray *et al.*, 1998). The present study, therefore, urges the need for conservation of the Asian tropical tree snails of the genus *Amphidromus* that exhibit whole-body enantiomorphy and dextral and sinistral monomorphisms in multiple extant species.

### Phylogenetic and taxonomical implications

*Beddomea* has been classified as a subgenus of *Amphidromus* (Pilsbry, 1901) and then raised to a distinct genus (Zilch, 1960). The present results show that *Beddomea* is closer to *Camaena* and *Chloritis* than *Amphidromus*, supporting the current taxonomy of *Beddomea*. Our mtDNA phylogeny also supports the taxonomical separation of the subgenus *Amphidromus* from another subgenus *Syndromus*. It implicates that their distinct differences in shell size, shape, and colour pattern and in genital structure reflect their evolutionary history. The present topology also supports the separation of *A. schomburgki* from *A. givenchyi* as distinct species by Zilch (1953) and Sutcharit & Panha (2006b).

*Amphidromus atricallosus perakensis* showed closer affinity with two other species than with conspecific taxa. *Amphidromus  atricallosus perakensis* differs from the supposedly conspecifics in shell colour pattern lacking a dark parietal callus and varices (Sutcharit & Panha, 2006b). The consistent presence and absence of varices in *A. atricallosus classiarius* and *A. atricallosus perakensis*, respectively, suggest the stability and usefulness of that character state for taxonomy. *Amphidromus  atricallosus perakensis* was formerly classified as a different species (Fulton, 1901), but has been a subspecies since Laidlaw & Solem (1961). *Amphidromus atricallosus perakensis* may be a separate species reproductively isolated from other populations in *A. atricallosus*. The current pattern could also result from introgression or ancestral polymorphism of mtDNA. The chance of interspecific hybridization would not be large because no sympatry has been found in the subgenus *Amphidromus*, although that does not preclude hybridization during the history of radiation.

The apparent paraphyly of *Syndromus* indicates that enantiomorphic *A. glaucolarynx* and sinistral *Syndromus* should be recognized as distinct subgenera. It is notable that the exceptional enantiomorphy of *A. glaucolarynx* in the current *Syndromus* reflects its phylogenetic history, rather than the secondary derivation of enantiomorphy in the sinistral clade. The values of bootstrap support around 60% (Fig. 2, see Figs S2 and S3) suggest that the basal split of *A. glaucolarynx* needs to be assured with a further analysis including a larger sample of taxa. However, our posteriori evaluation of morphology has supported the distinction of *A. glaucolarynx* from the rest of *Syndromus* in radula teeth, shell size, shape and colour pattern, and epiphallic caecum. On this account, additional studies of morphology would reveal synapomorphic characters of *Syndromus sensu stricto*.

The current taxonomy of sinistral species in *Syndromus* should also be revised based on further studies of anatomy and genetic divergence using nuclear DNA markers. We have detected polyphyletic origins of mtDNA haplotypes in each of three species: *A. semitessellatus*, *A. xiengensis* and *A. areolatus*. First, the three haplotypes of *A. semitessellatus*, all from Thailand, were divided by 22.5% base substitution into the two largest sinistral clades of *Syndromus*. One haplotype from the central (locality 11) Thailand exhibited affinity to those of two other species from the Malay Peninsula and Borneo. On the other hand, the other two haplotypes from Chanthaburi (localities 12 and 13) near Cambodia were close to *A. areolatus* from Pangnga (locality 21) on the Peninsula. Thus, these divergent haplotypes of *A. semitessellatus* are not simply ascribable to interspecific introgression, which is possible only in sympatry. Instead, two types of *A. semitessellatus* may represent genetically independent populations exhibiting closely similar shell-colour patterns. Secondly, the haplotypes of *A. xiengensis* were separated into three clusters, which are all supported by high bootstrap probabilities. One of those clusters includes *A. xiengensis* from an island near Bangkok (locality 15) and *A. porcellanus* from Java (locality 34), which could hardly be explained by mtDNA introgression considering their remote localities. Thirdly, one haplotype of *A. areolatus* was closer to that of *A. xiengensis* than the other conspecific haplotype, which was clustered with a haplotypes of *A. semitessellatus*, in spite of the sampling localities of *A. areolatus* all within a range across the Peninsula.

For these reasons, the polyphyly of mtDNA haplotypes repeatedly discovered within the three species of *Syndromus* poses intriguing questions on convergence and polymorphism of shell colour patterns. Shells of *Syndromus* commonly exhibit complicated mosaic colour patterns which are likely to camouflage arboreal snails from predators. Because of this critical function for survival, the colour patterns may have converged; or, diverging species may have maintained polymorphism of similar colour patterns. Thus, the polyphyly found in *Syndromus* implicates ecologically significant functions of shell colour pattern that is variable within and/or between species. The present results, however, cannot reject introgressive hybridization or ancestral polymorphism of mtDNA. Thorough analyses of phylogeography using nuclear as well as mtDNA markers and of shell colour patterns are necessary to resolve these issues.

## Conclusions

Enantiomorphy of the primary asymmetry has persisted within populations in the tree snail genus *Amphidromus* probably as the ancestral character state. It demonstrates that directional asymmetry is not the inevitable norm of developmental polarity in animal body plan. This study underscores the need for explicit studies of a mechanism responsible for the maintenance of whole-body enantiomorphy and for conservation of the last remaining enantiomorphic tree snails. The present evidence of enantiomorphy illuminates a fundamental question on the general rule: why is the primary asymmetry directional in other animals? Closer scrutiny of the genus *Amphidromus* may test the aphorism that ‘the exception proves the rule’.

## Supplementary Material

The following supplementary material is available for this article online:

Table S1 Names of sampling localities for mtDNA phylogeny.

Table S2 Names of localities and areas for enantiomorph frequency survey.

Figure S1 Neighbor-joining dendrogram.

Figure S2 Maximum likelihood dendrogram.

Figure S3 Contrasting mating behaviours of enantiomorphic Amphidromus atricallosus and sinistral A. xiengensis.

This material is available as part of the online article from http://www.blackwell-synergy.com
