# Supplementary material for: Evolution of whole-body enantiomorphy in the tree snail genus Amphidromus
Source: J Evol Biol. 2007 Mar;20(2):661–72. doi: 10.1111/j.1420-9101.2006.01246.x (PMC1920546; doi:10.1111/j.1420-9101.2006.01246.x)
Supplement: Table S1 — Names of sampling localities for mtDNA phylogeny. [file jeb0020-0661-t1.doc]

**Supplementary Material**

Table S1. Names of sampling localities for mtDNA phylogeny. Locality numbers correspond to those in Table 1 and Figs. 1a and 2.

1. Luang Pra Bang, Laos; 2. Mae Yom National Park, Phrae, Thailand; 3. Tam Chieng Dao, Chieng Dao, Chiengmai; 4. Phu Luang Wildlife Sanctuary, Loei; 5. Phu Lom Khao, Na Ku, Kalasin; 6. Ban Khok klang, Tao Ngoi, Sakonnakhon; 7. Savanakhet, Laos; 8. Phataem National Park, Ubonratchathani, Thailand; 9. Pong Phu Ron, Thong Pha Phum National Park, Kanchanaburi; 10. Sai Yok Noi Waterfall, Sai Yok, Kanchanaburi; 11. Khao Ang Rue Nai Wildlife Sanctuary, Chachoengsao; 12. Makok Waterfall, Plieu National Park, Chanthaburi; 13. Trong Nong Waterfall, Plieu National Park, Chanthaburi; 14. Kaeng Kracharn National Park, Petchaburi; 15. Elar Island, Sattahip, Chonburi; 16. Kud Island, Koh Kud, Trat; 17. Pra Kra Yang Cave, Kraburi, Ranong; 18. Hot Spring, Muang, Ranong; 19. Na Muang Waterfall, Koh Samui, Suratthani; 20. Ban Takun, Suratthani; 21. Muang, Pangnga; 22. Kra Island, Pak Panang, Nakhonsrithammarat; 23. Khao Poo-Khao Ya National Park, Patthalung; 24. Danum Valley, Sabah, Malaysia; 25. Sukau, Sabah, Malaysia; 26. Banggi, Sabah, Malaysia; 27. Natuna Island, Indonesia; 28. Tioman Island, Malaysia; 29. Sarawak, Malaysia; 30. Nee Soon, Singapore; 31. Botanical Garden, Singapore; 32. Kabupaten Lampung Selatan, Indonesia; 33. Pangandaran, Indonesia; 34. Bogor, Indonesia; 35. Pitakele, Sri Lanka.

Table S2. Names of localities and areas for enantiomorph frequency survey. Locality numbers correspond to those in Figure 1b and Tables 2 and 3. In several places, we surveyed along a trail or road because of the physical difficulties of surveying in quadrats.

b

c
